# Luetic aortopathy: Revisited

**DOI:** 10.4103/2589-0557.75011

**Published:** 2010

**Authors:** Bhushan Sevakram Madke, Nandkishor Babulal Agrawal, Pradeep Vaideeswar, Mayuresh Pradhan, Amey Vijay Rojekar, Uday Sharadchandra Khopkar

**Affiliations:** Department of Dermatology, Seth GS Medical College and KEM Hospital, Mumbai, Maharashtra, India; 1 Department of Cardiothoracic Surgery, Seth GS Medical College and KEM Hospital, Mumbai, Maharashtra, India; 2 Department of Pathology (Cardiovascular and Thoracic Division), Seth GS Medical College and KEM Hospital, Mumbai, Maharashtra, India

**Keywords:** Aneurysm, aortitis, coronary ostia, disseminated intravascular coagulation, syphilis, therapeutic paradox, therapeutic shock

## Abstract

We report a case of 38-year-old male, who presented with a large pulsatile swelling on the left side of the anterior chest wall of 4 months’ duration with a gradual increase in size. He gave history of sexual promiscuity in the form of unprotected sexual intercourse prior to his marriage in his early 20s. He also gave a history of ulceration on coronal sulcus of glans penis 20 years back with painless right inguinal mass. His blood serology was strongly positive for syphilis and hepatitis B surface antigen (HBsAg); however, serology for retroviral infection was negative. Computed tomography–angiography confirmed the pulsatile swelling as aneurysm of the arch of and ascending aorta. In view of the history, positive serology, and imaging studies, we concluded the aortic aneurysm to be of syphilitic origin. We report this case due to its extreme rarity in the present antibiotic era.

## INTRODUCTION

Syphilis is an infectious disease which if untreated occurs sequentially through various stages and remains latent for many years.[1] Approximately, 30% of untreated syphilitic patients present with a tertiary stage after a latent period ranging from 10 to 30 years.[2] Cardiovascular disease can be divided into four categories: (1) uncomplicated syphilitic aortitis; (2) syphilitic aortic aneurysm; (3) syphilitic aortic valvulitis with aortic regurgitation; and (4) syphilitic coronary ostial stenosis.[3] Ascending (50%) and arch of aorta (10–15%) are commonly involved in the syphilitic process,[4] weakening the thick muscular wall and giving rise to saccular aneurysm. If not treated timely, it can lead to complications like spontaneous rupture and dissection thereby leading to exsanguination culminating in instantaneous death.[5] We report a case of large syphilitic aortic aneurysm eroding the sternum which was treated surgically, but unfortunately the patient died on the first postoperative day.

## CASE REPORT

A 38-year-old married male working in a chemical factory presented to our outpatient department with a large pulsatile swelling of 4 months’ duration on the left side of the chest. Initially, he noticed a small 4 × 4 cm swelling below the left clavicular region, which rapidly increased in size to about 10 × 10 cm in 4 months. He complained of breathlessness on climbing a normal flight of stairs. He also had a dragging sensation in the chest and hoarseness of voice since last 2 months. There was no history of preceding trauma. On enquiry, the patient gave history of sexual promiscuity in the form of multiple unprotected heterosexual intercourses with multiple partners prior to his marriage in his early twenties. The patient also gave history of painless ulcer on coronal penis with painless inguinal swelling about 20 years back following exposure for which he did not seek any medical advice. Inguinal swelling was treated by incision and drainage by a local doctor. The patient denied any history of skin rash on his body. There was no history of any neurological problem. His personal history revealed alcohol abuse and smoking.

**Figure 1:**
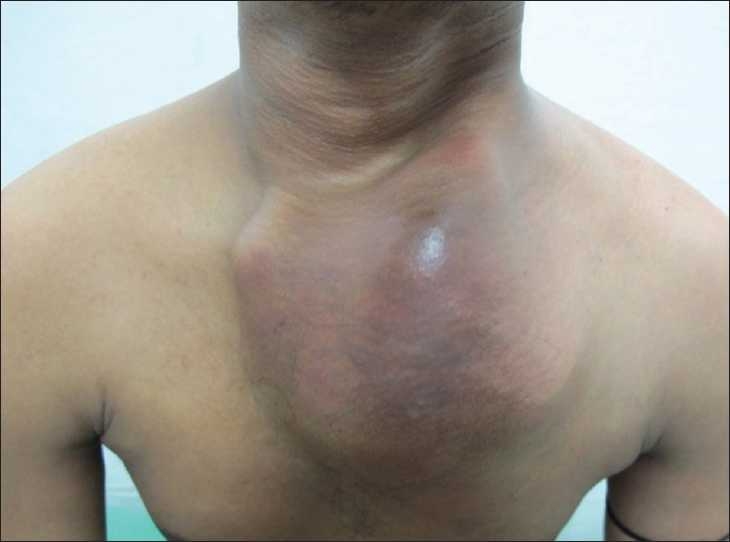
A large swelling on the left side of the anterior chest wall

**Figure 2:**
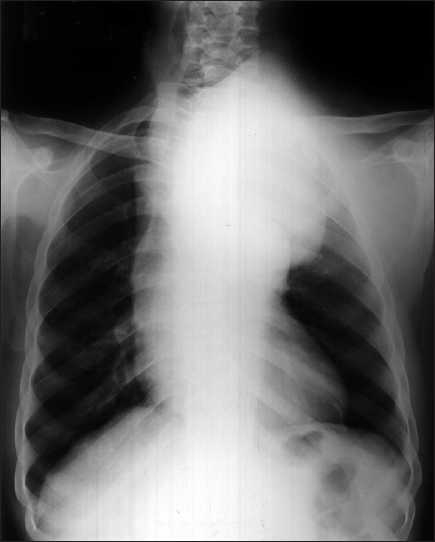
Chest roentgenogram showing widening of the mediastinum with a radiopaque mass extending into the superior mediastinum occupying the left upper and mid-zone with a right tracheal shift

**Figure 3:**
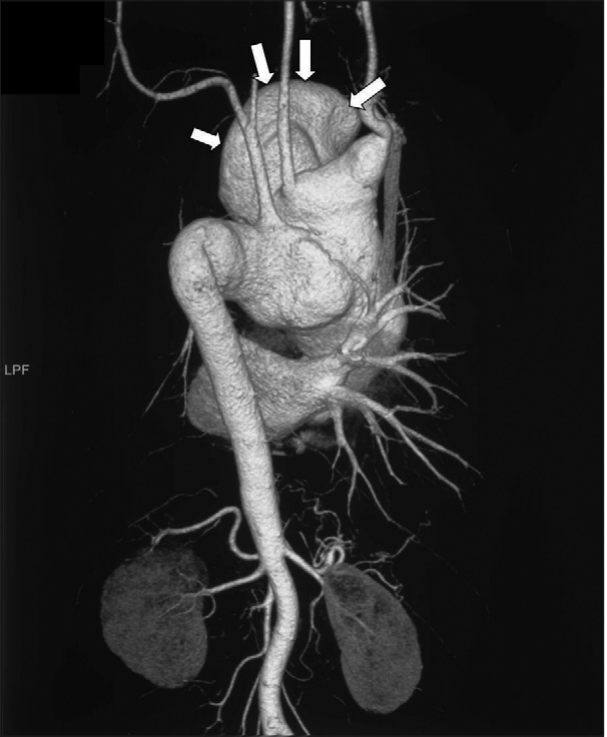
Three-dimensional reconstructive image of the cardiac angiogram showing aneurysm of the arch of and ascending aorta indicated by arrows

Local examination showed a large well-defined pulsatile swelling on the left side of the anterior chest wall measuring about 10 × 10 cm externally [Figure 1]. Palpation confirmed a pulsatile swelling with bruit in some areas associated with auscultatory thrill. His general and systemic examinations including neurological examination were within normal limits. His height and weight were 156 cm and 42 kg, respectively. Cutaneous, genital, and mucosal examinations were negative for any syphilitic stigma except for the scar in the right inguinal region which corroborated with the past history of an inguinal bubo. His hemogram revealed a low hemoglobin of 9.0 g/dl with a normal white blood cell count of 5600/mm^3^. Serum chemistry (blood urea nitrogen 16 mg/dl, serum creatinine 0.8 mg/dl, aspartate transaminase [AST] 22 U/l, alanine transaminase [ALT] 13 U/l) including serum lipid levels and urinalysis was within a normal range. His blood serology was positive for syphilis (Venereal Disease Research Laboratory test [VDRL] 1:32 and *Treponema pallidum* haemagglutination assay [TPHA] 1:2560) and hepatitis B surface antigen (HBsAg); however, serology for human immunodeficiency virus infection was negative. The VDRL of the cerebrospinal fluid was nonreactive. Ophthalmic examination did not show any signs of papilledema. Electrocardiogram showed normal sinus rhythm with no evidence of left ventricular hypertrophy. His blood pressure was 180/70 mm of Hg in right upper and lower extremity. Chest roentgenogram showed widening of the mediastinum and a mass in the left upper and mid-zone with a marginal right shift of the trachea [Figure 2]. Computed tomography (CT)–angiography revealed a saccular aneurysm of the ascending and arch of aorta of an approximate size of 8 cm penetrating through the chest wall in the left parasternal region and peripheral thrombus of 4 cm with a bony erosion of the sternum and first three ribs on the left side; however, origins of coronary arteries were spared. A three-dimensional reconstruction image of the coronal view of cardiac angiography showed aneurysm of the ascending and arch of aorta along with arch vessels [Figure 3]. Abdominal sonogram showed a mild degree of hepatomegaly which was consistent with the carrier stage of serum hepatitis. Two-dimensional echocardiography of the heart showed the following findings: left atrium 2.8 cm; aortic annulus 1.9 cm; left ventricular internal dimension 2.8 cm (systole); left ventricular internal dimension 3.9 cm (diastole); left ventricular septum 1.1 cm; left ventricular posterior wall 1.1 cm; ejection fraction 60%, and mild aortic regurgitation with aneurysm mainly involving the ascending and arch of aorta.

**Figure 4:**
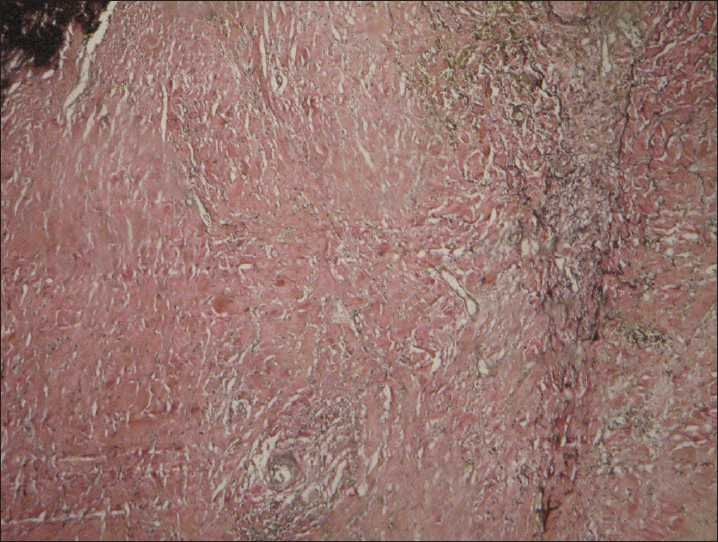
Elastic Van-Gieson stain specimen showing the wall of aneurysm composed of fibrohyaline tissue with fragmented elastic fibers (magnification, ×40)

The patient was immediately admitted to the cardiothoracic surgery division for definitive management. Penicillin treatment given for syphilis can lead to thrombotic complications (due to the local antigen release); hence, the prophylactic course of oral steroids in the form of prednisolone 20 mg daily was advised. After a week of oral steroids; the patient was given injectable benzathine penicillin G 2.4 million units once a week for three consecutive weeks after intradermal sensitivity testing. The steroid cover was continued for a period of 6 weeks with a slow taper following penicillin therapy. Aneurysm was replaced with a synthetic albumin-coated Dacron graft under cardiopulmonary bypass using the technique of hypothermic circulatory arrest at 20–22°C/68°F. Cerebral circulation was maintained through the antegrade flow via right carotid artery cannulation. However, the patient continued to bleed through the chest tube during the postoperative period and was found to have disseminated intravascular coagulation secondary to hypothermia and multiple blood transfusions (D-dimer levels were raised). Unfortunately, the patient succumbed to death on the first postoperative day. Histopathology of the postmortem diseased aortic segment showed changes suggestive of syphilitic pathology on special stain for elastin in which elastic fibers were found to be degenerated and fragmented [Figure 4].

## DISCUSSION

Our patient had two sexually acquired infections, i.e., syphilis and hepatitis B, reflecting his high-risk sexual behavior. In view of the involvement of the ascending aorta and positive serology for syphilis in the absence of systemic hypertension and significant atherosclerosis, we diagnosed this case as luetic aortopathy. Cases of the tertiary stage in the form of cardiovascular syphilis still come into light in 30% of untreated patients.[4] The widespread use of antibiotics for treatment of various body infections has made cardiovascular syphilis a medical curiosity.[6] Atherosclerosis remains the main cause of aneurysm of thoracic and abdominal vessels although one should always keep in mind the possibility of luetic aortitis in patients under 50 years of age having cardiac symptoms and showing no signs of atherosclerotic vascular disease.[7] Approximately, 90% of syphilitic aneurysms are located in the ascending aorta or arch of aorta.

Treponema invades the vasa vasorum (minute vessels supplying great vessels) of aorta and elicits an inflammatory reaction consisting of lymphoplasmacytic and perivascular infiltrates that lead to endarteritis obliterans, adventitial scarring, and patchy necrosis of the medial layer with elastic fiber destruction.[2] The damaged intima then becomes a nidus for atherosclerotic patches. The ascending and arch of aorta bear the brunt of the disease process and all these pathological changes lead to weakening of the vessel wall, leading to loss of vascular elasticity resulting in aneurysm and dilatation.

Clinical suspicion of syphilitic aortitis can be made when loud and tambour-like second heart sound (*bruit de tabourka*) is heard in a patient who has neither hypertension nor atherosclerosis.[8] Palpatory bruits may be absent in aneurysm if there is a presence of intramural thrombus. Symptoms of luetic aortic aneurysm vary and include pulsatile anterior chest wall mass, palpitations, dyspnea, chest pain radiating to back, cough, and hemoptysis. In syphilitic aortitis, where adventitia of great vessels (richly innervated by nerves) is inflamed can lead to chest pain and can simulate angina pectoris due to myocardial ischemia. Syphilitic aortic root dilatation and subsequent insufficiency can cause left ventricular eccentric hypertrophy thereby leading to massive heart enlargement (weight more than 1000 g) and termed as cor bovinum (cow’s heart).[9] Aneurysm can cause compressive symptoms, such as dysphagia, dyspnea, hoarseness, cough, and superior vena cava compression syndrome.[10] Complications of aortic aneurysm include rupture, bleeding, thrombus formation with distal embolization (cerebral, visceral, or limb), aortic dissection, septic embolization, aortic insufficiency, and acute coronary syndromes caused by coronary involvement.[10] Our patient complained of grade III dyspnea (due to compression of trachea and both bronchi by aneurysm) and hoarseness of voice due to compression of the left recurrent laryngeal nerve (Ortner’s syndrome).[11] Dysphagia caused by similar compressive mechanism is called as “dysphagia aortica.” Aortic aneurysm can also cause persistent cough as a result of pressure on the recurrent laryngeal nerve thereby triggering the cough reflex.

A CT scan with contrast enhancement is the imaging technique of choice.[12] It allows rapid exclusion of other aortic pathologies that may sometimes resemble acute aortitis, such as aortic dissection, intramural hematoma, and penetrating atherosclerotic ulcer.

However, extreme caution has to be paid while administering penicillin in cardiovascular syphilis as it can lead to Jarisch-Herxheimer reaction, which along with systemic signs and symptoms of fever headache and malaise flushing (therapeutic shock) can cause acute aortic wall inflammation with abrupt closure of the coronary ostia (therapeutic paradox).[13,14] Myocardial infarction is a dreaded complication if the luetic process has affected the coronary ostia, and after penicillin therapy, the local release of antigens can cause occlusion of the coronary ostia due to inflammation and *in situ* thrombosis. In our patient, the origin of coronary arteries was spared.

The prophylactic administration of systemic steroids lessens considerably the incidence of Jarisch- Herxheimer reactions in cardiovascular syphilis.[15] Harboring aneurysm of more than 6-cm diameter is like a live time bomb, which requires urgent surgical repair with placement of a prosthetic graft. In our case, the patient was unable to withstand the operative procedure and succumbed to hemodynamic instability secondary to disseminated intravascular coagulation and blood loss from the anastomotic site within few hours of surgery. Disseminated intravascular coagulation is a known complication of complicated aortic aneurysm and cardiopulmonary bypass.[16] Hypothermia induced for the circulatory arrest has been shown to impair the activity of the enzymes involved in the platelet activation pathways and to reduce the enzymatic activity of clotting factors upon coagulation activation.[17] Multiple blood transfusion leads to the dilution of coagulation factors which can lead to consumptive coagulopathy as happened in our patient.
